# New resampling method for evaluating stability of clusters

**DOI:** 10.1186/1471-2105-9-42

**Published:** 2008-01-24

**Authors:** Irina M Gana Dresen, Tanja Boes, Johannes Huesing, Markus Neuhaeuser, Karl-Heinz Joeckel

**Affiliations:** 1 Institut für Medizinische Informatik, Biometrie und Epidemiologie, Universitaetsklinikum Essen, Germany; 2 Koordinierungszentrum für Klinische Studien, Universitaetsklinikum Heidelberg, Germany; 3 Fachbereich Mathematik und Technik, RheinAhrCampus Remagen, Germany

## Abstract

**Background:**

Hierarchical clustering is a widely applied tool in the analysis of microarray gene expression data. The assessment of cluster stability is a major challenge in clustering procedures. Statistical methods are required to distinguish between real and random clusters. Several methods for assessing cluster stability have been published, including resampling methods such as the bootstrap.

We propose a new resampling method based on continuous weights to assess the stability of clusters in hierarchical clustering. While in bootstrapping approximately one third of the original items is lost, continuous weights avoid zero elements and instead allow non integer diagonal elements, which leads to retention of the full dimensionality of space, i.e. each variable of the original data set is represented in the resampling sample.

**Results:**

Comparison of continuous weights and bootstrapping using real datasets and simulation studies reveals the advantage of continuous weights especially when the dataset has only few observations, few differentially expressed genes and the fold change of differentially expressed genes is low.

**Conclusion:**

We recommend the use of continuous weights in small as well as in large datasets, because according to our results they produce at least the same results as conventional bootstrapping and in some cases they surpass it.

## Background

Cluster analysis is a widely used tool for interpretation of gene expression experiments. It allows to group genes as well as (tissue) samples in classes (clusters) of similar characteristic profiles. Class assignment results from applying a similarity measure (i.e. distance measure or correlation) and a selected method to calculate the distance of an object to a class (i.e. single, complete or average linkage). The algorithms are well-defined and reproducible, however the choice of different similarity measures and cluster methods leads to different results [1].

Algorithms for hierarchical agglomerative classification exist for a long time [2-6]. They are suitable for the description of highly dimensional data. Eisen et al. introduced hierarchical cluster analysis for microarray data in 1998 [7].

A problem in cluster analysis is to discriminate between real and random clusters. The latter arise from random variation of gene expression measurements due to technical variation and biological variability. A measure of cluster stability is needed to solve this problem.

Several methods for validating clusters internally have been described [8-19]. The basic idea is to apply the same methods to data similar to experimentally derived data or that "might as well" have been generated. One idea is to add a (normally distributed) error term on all measurements [8,9]. Thalamuthu et al. [15] perturb simulated data to evaluate and compare gene clustering methods in microarray analysis. Another method depends on the study of random samples from the original dataset. Smolkin and Ghosh [10] use this method to assess the stability of clusters in hierarchical cluster analysis of microarray experiments. They calculate a cluster stability score as a percentage of how often a cluster occurs in the samples. Monti et al. [16] propose a consensus clustering where multiple runs of a clustering algorithm are performed on subsampled data and a consensus across these is determined. Tseng and Wong [17] use a different approach to identify stable clusters. They iteratively apply k-means to subsamples of the original data and use the results as classifiers to cluster the original data. A review on clustering validation is given by Handl et al. [14]. They distinguish between external and internal measures. Whereas external validation measures require additional knowledge of class labels, internal validation techniques are only based on the information intrinsic to the data alone. The Rand Index [20,21] which determines the similarity between two partitions is an example for an external validation measure. Internal measures comprise different types of validation techniques. Types referring to the particular notion of clustering quality that they employ assess compactness, connectedness and separation of clusters or a combination of these. A different class of methods is to repeatedly resample or perturb the original dataset and re-cluster the resulting data. Nearest-neighbor based methods, the bootstrap and our proposed method belong to this class. An alternative method is to estimate the degree to which distance information in the original data is preserved in a partitioning. Finally there exist specialized measures for highly correlated data, such as the figure of merit. Datta and Datta [18,19] compute a figure of merit based on three validation measures:, an average proportion of non-overlap, an average distance between means and an average distance. All of them are computed under consideration of the full data and the data obtained by deleting the expression levels at one time point at a time. These values are expected to be small for a good clustering algorithm.

Our proposed method is similar to the bootstrap. Only a few applications of the bootstrap method in cluster analysis over arrays can be found in the literature. Zhang and Zhao [11] use the bootstrap for hierarchical cluster analysis. They summarize individual dendrograms in a consensus-tree. Their method requires estimates for the impreciseness of gene expression measurements.

Also Bhattacharjee et al. [22] use bootstrapping to assess clustering stability and to validate the results output by hierarchical clustering.

Kerr and Churchill [12] use the bootstrap for assessing the stability of the results of cluster analyses. It is based on an ANOVA model to estimate the relative gene expression and to consider other sources for variation of microarray data. The percentage of genes in bootstrap clusters is a measure for assessing the stability.

Dudoit and Fridlyand [13] use bagging (bootstrapping and aggregation) to improve the accuracy of a partitioning cluster method. The individual partitions are combined to one final partition or a new dissimilarity matrix is built and serves as basis of the final classification.

As bootstrapping is a drawing with replacement and the size of the bootstrap sample is the same as the original data size, some observations are omitted. The expected proportion of points in the original sample absent from the bootstrap sample is given by (1 - 1/*n*)^*n *^[23], which converges to 1/e for n → ∞, or approximately 36.8 per cent. We propose the use of continuous weights instead of bootstrap. Continuous weights avoid zero elements and instead allow non-integer weights and thus every observation is represented in the resampled dataset.

Several methods for combining individual dendrograms to a consensus tree exist. The majority rule consensus tree [24], which only considers nodes that are present in at least 50% of the dendrograms, is among the most often applied consensus trees.

Two partitions can be compared by application of a similarity measure such as the Rand index [20,21]. In case of the existence of scattered genes Thalamuthu et al. [15] propose a weighted Rand index.

Here we report of a new resampling method that is based on continuous weights. The creation of resampled datasets based on weighted sampling is similar to the creation of bootstrap samples but instead of drawing whole observations, random floating-point numbers larger than zero are drawn and the observations are weighted by these numbers so that each observation is represented in the resampled dataset. We compare this method to the conventional bootstrap to show where it is advantageous.

## Results

We compared continuous weights and the conventional bootstrap using real microarray gene expression data as well as simulated data. Real data were preprocessed as specified in the corresponding papers. Majority rule consensus trees were generated from the individual dendrograms derived from continuous weights or bootstrap. For each combination original dendrogram/consensus tree obtained by weight matrix or bootstrap the weighted Rand index was calculated.

### Real datasets

We used two real datasets for evaluating our new method and comparing it to the bootstrap. The first dataset was the uveal melanoma dataset of Tschentscher et al. [25]. In this dataset gene expression was measured in 20 patients with uveal melanoma. Ten patients had a normal chromosome set and the other ten showed a monosomy of chromosome 3. This dataset was divided into 24 small datasets according to the chromosomal location of the genes. Hierarchical clustering was done with average linkage.

Comparing continuous weights and bootstrap revealed for these datasets that in three cases (chromosomes 1, 9 and 19) the clustering was exactly the same (Table 1). Only some clusters were drawn with higher frequency when utilizing continuous weights. In 13 cases the clustering showed minimal differences but overall the results for weighted sampling and bootstrap were similar. However in eight cases the clustering was considerably different. In these cases continuous weights always led to more informative results than bootstrap because more reliable clusters could be identified.

**Table 1:** Clustering of uveal melanoma datasets [25] with continuous weights and bootstrap

**chromosome**	**number of probe sets**	**identical**	**minimal difference**	**considerable difference**
1	648	X		
2	406			X
3	336			X
4	200		X	
5	288		X	
6	416			X
7	298			X
8	222		X	
9	218	X		
10	238		X	
11	372		X	
12	361			X
13	120		X	
14	198			X
15	186		X	
16	268		X	
17	393		X	
18	102		X	
19	393	X		
20	163		X	
21	80		X	
22	205			X
X	285		X	
Y	8			X

The marks in the table denote whether the obtained consensus trees are identical or show minimal or considerable differences.

**Figure 1:**
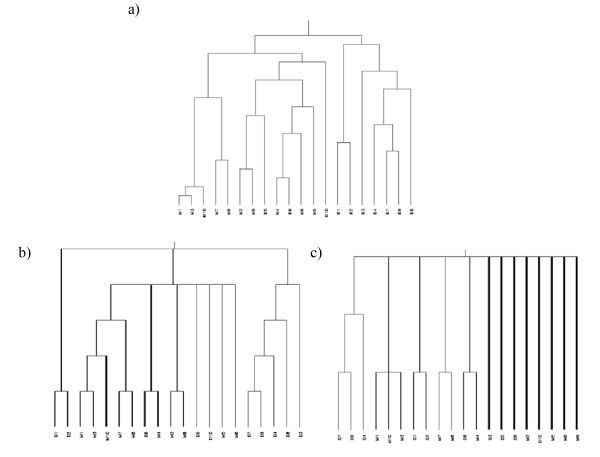
**Hierarchical clustering of chromosome 6 (412 probe sets) from the uveal melanoma data**. a) original dendrogram, b) consensus tree continuous weights, c) consensus tree bootstrap.

Figure 1a) shows the original clustering of chromosome 6. Patients are abbreviated as D1–D10 for patients with disomy 3 and M1–M10 for patients with monosomy 3. Two large clusters can be seen. One of them contains only patients with a normal karyotype. The other one comprises all patients with monosomy 3 plus three patients with normal karyotype. Bootstrapping on the patients leads to uninformative results because reliable clusters cannot be determined (Rand index = 0.041) (Figure 1b). Eight patients cannot be assigned to any cluster and thus only appear clustered on the top level. On the other hand utilization of continuous weights nearly reproduces the original dendrogram (Rand index = 0.231) (Figure 1c). The left and right cluster of the consensus tree match exactly the right cluster of the original dendrogram which contains only patients without monosomy 3 and the members of the cluster in the middle of the consensus tree can all be found in the left cluster of the original dendrogram. Unfortunately the true classification in this case is unknown.

**Figure 2:**
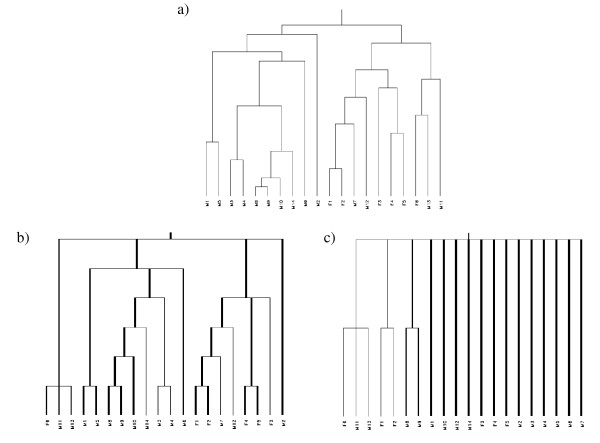
**Hierarchical clustering of chromosome Y (8 probe sets) from the uveal melanoma data**. a) original dendrogram, b) consensus tree continuous weights, c) consensus tree bootstrap.

In the case of the Y-chromosome (Figure 2) the true classification is known. The dataset consisted of 14 men and 6 women. Patients are abbreviated as M1–M14 for male patients and F1–F6 for female patients. All women cluster together in one cluster. Due to the fact that some of the probe sets show cross-hybridization 4 men cluster together with the women. Only when utilizing continuous weights this cluster behavior is shown correctly (Rand index = 0.137). Again with bootstrap no meaningful reliable clusters can be identified (Rand index = -0.036). Twelve patients are not assigned to a cluster except the cluster containing all patients. This may be due to the small number of probe sets.

The whole uveal melanoma dataset was clustered as well. Probably due to the large number of genes only minimal differences between continuous weights matrices and bootstrap were found (data not shown).

Next, continuous weights and bootstrap were compared using a dataset where seven features, i.e. the maximal age of death and some birth and pregnancy data, were measured in 22 primates [26,27]. Hierarchical clustering was done with the complete linkage method.

**Figure 3:**
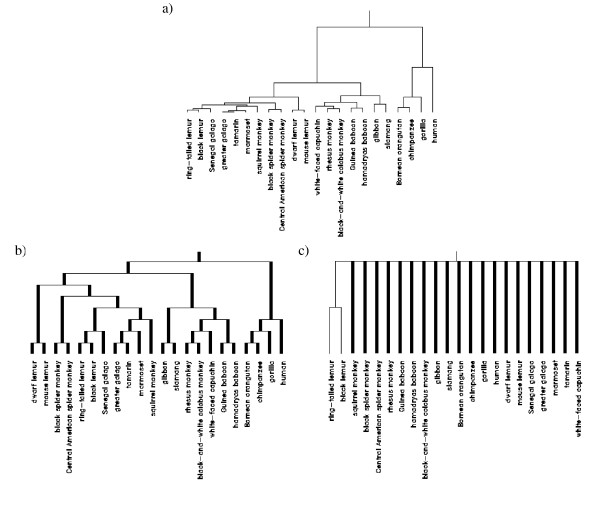
**Hierarchical clustering of primate data (7 features) [26,27]**. a) original dendrogram, b) consensus tree continuous weights, c) consensus tree bootstrap.

Results are shown in Figure 3. Once more nearly all primates cannot be assigned to a cluster when using bootstrap (Rand index = -0.071) whereas with continuous weights the original dendrogram is exactly reproduced (Rand index = 1). This classification is quite reasonable because it reproduces the taxonomy of the old world apes, with the exception of the white-faced capuchin (*Cebus capucinus*), and their separation from the half-and-half apes and the new world monkeys. The other families and subfamilies are not replicated. The failure of bootstrap again could result from the small number of features in the data set.

### Simulated data

Simulation studies uncovered relationships between the cluster behavior and the number of differentially expressed genes, the number of observations, the size of the groups, the fold change and the number of groups respectively.

**Figure 4:**
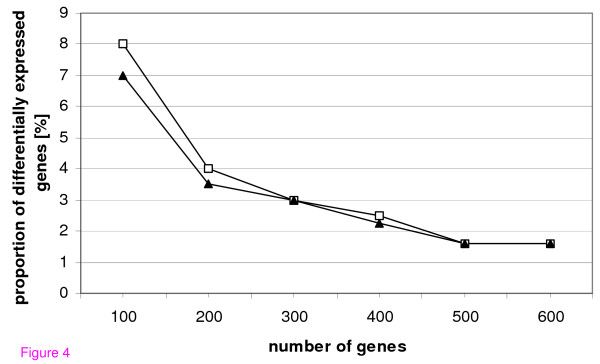
**Simulation study**. Two groups with 10 variables each, fold change of 9, number of genes and number of differentially expressed genes vary, symbols indicate the proportion of differentially expressed genes where groups are just not separated any more; □: bootstrap, ▲: continuous weights

In the first simulation (Figure 4) data for two groups with 10 variables each were generated by choosing a constant fold change of 9 and varying the number of genes and the number of differentially expressed genes. The proportion of differentially expressed genes where groups are just not separated any more was analyzed. The smaller the number of genes the better is the performance of continuous weights over bootstrapping. Using 500 genes continuous weights and bootstrapping show no differences.

**Figure 5:**
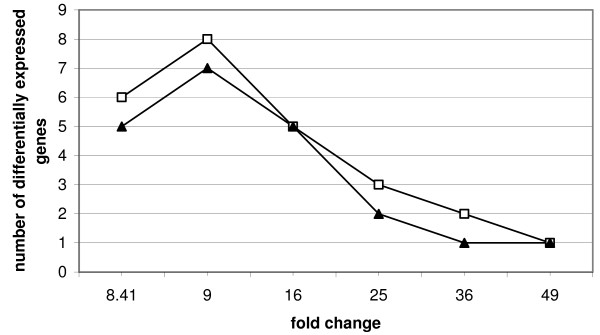
**Simulation study**. Two groups with 10 variables each, number of genes equals 100, fold change and number of differentially expressed genes vary, symbols indicate the number of differentially expressed genes where groups are just not separated any more; □: bootstrap, ▲: continuous weights

In the next simulation (Figure 5) the settings were the same as in the first simulation except the number of genes was kept constant at a value of 100 and the fold change was varied between 9 and 49. Again the number of differentially expressed genes where groups are just not separated any more was analyzed. No difference between continuous weights matrices and bootstrapping could be detected if the fold change was larger than about 40. For a fold change smaller than 8.41 the groups were not separated any more even in the original dendrogram when the number of differentially expressed genes was in the range, which was relevant for differences between weighted sampling and bootstrapping. If the fold change was between 8.41 and 36 less differentially expressed genes were needed for continuous weights than for bootstrapping to separate the groups.

**Figure 6:**
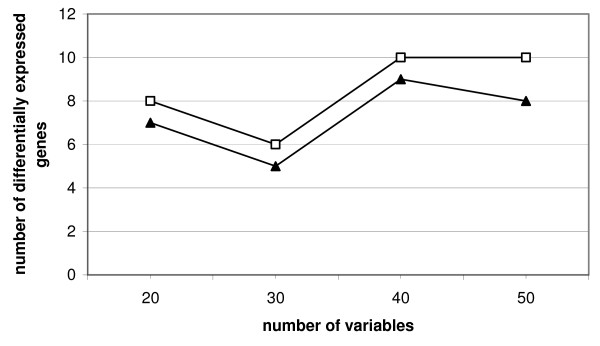
**Simulation study**. Two groups, number of genes equals 100, fold change of 9, number of variables and number of differentially expressed genes vary, symbols indicate the number of differentially expressed genes where groups are just not separated any more; □: bootstrap, ▲: continuous weights

In another simulation the number of genes was kept constant at 100 genes, the fold change was constantly 9 and the number of differentially expressed genes was varied. The number of variables was varied between 10 and 25 per group with both groups having the same size resulting in 20 to 50 variables (Figure 6). As in the other simulations the number of differentially expressed genes where groups are just not separated any more was analyzed. The advantage of continuous weights over bootstrapping can be easily seen, as up to 25 variables per group (50 total) less differentially expressed genes are needed to separate the groups.

**Figure 7:**
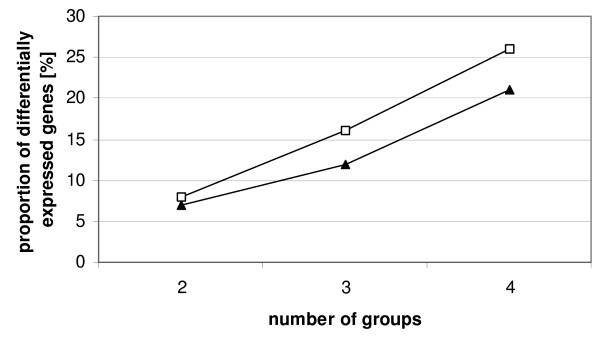
**Simulation study**. Number of genes equals 100, fold change constant, number of groups and number of differentially expressed genes vary, symbols indicate the number of differentially expressed genes where groups are just not separated any more; □: bootstrap, ▲: continuous weights

In yet another simulation the number of groups was increased to three or four groups with an equal number of variables in each group (Figure 7). The number of genes was kept constant at 100 genes and the number of differentially expressed genes was varied. The fold change was kept constant as well but the value cannot be specified easily (see methods section). Again continuous weights performed better than bootstrapping although separation of groups was not as perfect as in the other simulations described above. The figure shows that the higher the number of groups the more differentially expressed genes were necessary to separate the groups both with continuous weights and bootstrapping. Also it is obvious that with increasing number of groups considerably less differentially expressed genes are needed to separate the groups with continuous weights than with bootstrapping.

## Discussion and Conclusion

Hierarchical clustering is an important explorative tool in microarray data analysis. It is often applied to get a first impression of the data structure of microarray gene expression experiments. It is important to assess the reliability of the clusters because random clusters may lead to a false interpretation of the data. Bootstrapping is one of the methods, which is used to determine the cluster stability. Nevertheless sometimes the results of bootstrapping are rather uninformative especially if the number of features is small. As about one third of the original observations is omitted in a bootstrap sample this finding is not surprising. Therefore we developed an alternative method similar to the bootstrap based on continuous weights, which have the advantage that every observation of the original dataset is retained in the resampled dataset. We hoped that this fact should improve the results of the conventional bootstrap. We evaluated our new method by real and simulated data and compared it to the bootstrap. The weighted Rand Index was applied to compare two partitions obtained by hierarchical clustering. By means of real data we could show that the continuous variant leads to more meaningful results than the conventional bootstrap when the number of features is small, and fares comparably when the number of features is large (as in many data sets obtained from microarray experiments).

Analysis of the 24 datasets of the uveal melanoma revealed that in most cases the same or nearly the same consensus trees are obtained using continuous weights and bootstrap. Sometimes the frequency of the clusters in the samples is higher when weights are used.

In some cases continuous weights led to more accurate information about cluster membership of individual observations. In the case of chromosome Y we made use of biological knowledge, i.e. the sex determines to which cluster an observation belongs, to confirm the true classification. We could show that only clustering methods based on weighted correlation distances are able to detect this.

Also with the primate dataset continuous weights outperformed the bootstrap. Again this result was expected because the dataset is very small.

The size of the dataset seems to be one of the important criteria for the advantage of weights over bootstrap. Especially in small datasets it is very important to consider every observation.

Simulation studies confirmed the benefits of continuous weights over bootstrapping. The new method is especially advantageous the smaller the number of genes and, above a threshold, the smaller the fold change. Of course the fold changes we used for simulation studies are not at all realistic for microarray gene expression data where already a fold change of two denotes differentially expressed genes. Yet in our settings we had to use such high fold changes to see any differences between continuous weights and bootstrapping. In real microarray gene expression data there are other factors such as the dependence and high correlation of microarray data that make continuous weights act better than bootstrapping.

Our results indicate that more simulation studies would be helpful to characterize the merits of continuous weights compared to the bootstrap. Simulated datasets should mimic microarray data sets more realistically to better understand the advantages of continuous weights.

Computing times are comparable as both bootstrapping and sampling weights have O(n) computing times, as have the calculation of the correlation coefficient in the weighted and unweighted variant, and every subsequent step was carried out in the same fashion with either method.

Nevertheless the use of continuous weights is strongly recommended because they perform at least as well as the bootstrap and in some cases they even surpass it.

It may be promising to study if in methods, which use the bootstrap as a part of it, a substitution of the bootstrap by our proposed method could improve the results. Methods coming into consideration are those from Kerr and Churchill [12] or Dudoit and Fridlyand [13]. Also integration of existing biological knowledge such as in Datta and Datta [28] should be possible to integrate. These approaches would of course require further studies.

Up to now we can only apply continuous weights in combination with the Pearson correlation. We plan to adapt the spearman correlation accordingly.

Furthermore we want to extend the application of continuous weights to other fields where bootstrap is employed such as k-means.

## Methods

For comparing the new method and the bootstrap real and simulated dataset were used. Real data were normalized according to the methods described in the corresponding papers. The uveal melanoma dataset of Tschentscher et al. [25] was divided into 24 smaller datasets according to the chromosomal location of the genes.

The creation of random continuous weights requires the generation of a suitable probability distribution function for the weights. The following considerations are useful for this purpose: if a procedure analogue to the bootstrap is applied on the level of variables it is equivalent to the usage of a weight vector for the original data whose elements are realizations of a multinomial distribution. An alternative procedure for bootstrap is the application of a weight vector with non-zero elements permitting non-integer diagonal elements. Thus the full dimensionality of space is maintained.

The underlying distribution of a drawing with replacement is the binomial distribution. This approximates to the Poisson distribution for a large number of observations and a small probability of success. Thus in a bootstrap sample the asymptotic distribution of the number of drawings per variable is Poisson(1) i.e. the expected value and variance is 1. To attain comparability with the bootstrap, the distribution from which weights for the weighted sampling are drawn has to have similar characteristics as the Poisson distribution but has to be continuous to allow every value for the diagonal elements. No continuous generalization of the Poisson distribution exists. Thus the distribution in demand has to have an expected value and variance of one and has to be positive. This is true for e.g. the lognormal distribution where -2μ = σ^2 ^with μ and σ as mean and standard deviation of the variable's logarithm. We used this distribution with μ = -log2 and σ^2 ^= 2*log2 deliberately as basis for drawing of the weights to attain the desired mean and variance. For each resampled dataset each observation from the original dataset was assigned a random weight, a correlation matrix was computed using the weighted Pearson correlation as similarity measure, i.e. the correlation coefficient was obtained by the formula

$$r_{ij}=\frac{\sum_{k}w_{k}(x_{ik}-{\bar{x}}_{i})(x_{jk}-{\bar{x}}_{j})}{\sqrt{\sum_{k}w_{k}{\left(x_{jk}-{\bar{x}}_{i}\right)}^{2}\sum_{k}w_{k}{\left(x_{jk}-{\bar{x}}_{i}\right)}^{2}}}$$

(where *x*_*ik *_denotes the kth feature on the ith specimen.) and the distance matrix was generated using the transformation *d*_*ij *_= 1 - *r*_*ij*_. Resampled datasets were clustered hierarchically using average or complete linkage, where all patients had initially the same weight (note that the weighting was used for genes and not patients). Individual dendrograms were summarized in a majority rule consensus tree according to published methods [24]. The thickness of the vertical lines denotes the frequency of the cluster in the consensus tree. For this purpose the frequency between 50 and 100 percent is divided into five equidistant disjoint classes and these are converted into the thickness of the lines in a linear relationship. The bootstrap method was applied to the same datasets using Pearson correlation as similarity measure for hierarchical clustering and the obtained dendrograms were summarized in a consensus tree as well. In each case 1000 resampled datasets were drawn.

The weighted Rand index [15], which is an extension of the adjusted Rand index of Hubert and Arabie [20] considering scattered objects, i.e. objects not being clustered, determined the measure of concordance between the consensus trees obtained by using continuous weights and bootstrapping and between the original dendrogram and the respective consensus trees. The adjusted Rand index is given by the formula

$$\text{Rand}=\frac{\sum_{i=1}^{R}\sum_{j=1}^{C}\left(\begin{array}{c} n_{ij}\\ 2 \end{array}\right)-\sum_{i=1}^{R}\left(\begin{array}{c} n_{i•}\\ 2 \end{array}\right)\sum_{j=1}^{C}\left(\begin{array}{c} n_{•j}\\ 2 \end{array}\right)/\left(\begin{array}{c} n\\ 2 \end{array}\right)}{0.5\left[\sum_{i=1}^{R}\left(\begin{array}{c} n_{i•}\\ 2 \end{array}\right)+\sum_{j=1}^{C}\left(\begin{array}{c} n_{•j}\\ 2 \end{array}\right)\right]-\sum_{i=1}^{R}\left(\begin{array}{c} n_{i•}\\ 2 \end{array}\right)\sum_{j=1}^{C}\left(\begin{array}{c} n_{•j}\\ 2 \end{array}\right)/\left(\begin{array}{c} n\\ 2 \end{array}\right)}$$

The weighted Rand Index is especially designed to compare different partitions of which at least one contains scattered objects. It consists of two parts, each of which is a slight modification of the adjusted Rand Index. The first part treats scattered objects as regular clusters, the second part ignores all scattered objects in either partition and thus is only based on intersection of clustered objects of both partitions. Finally both parts are averaged regarding the weights of the two measures.

Simulation was done by constructing two groups and drawing observations from the normal distribution. Differential expression was simulated by multiplication of a predefined number of observations with a factor in one group and division through this factor in the other group resulting in a fold change with the squared factor as value. Several simulations were performed, varying either the number of observations, the fold change or the size of the groups. In further simulations three or four groups were constructed. Differential expression for two of these groups was simulated as above. Additional groups were simulated by adding a multiple of the factor to a predefined number of observations. Thus a fold change cannot be specified easily any more.

All analysis was performed with SAS (version 9.1, SAS Institute Inc.).

## Authors' contributions

IGD participated in the design of the study, carried out the studies and drafted the manuscript. TB contributed to the design of the study. JH conceived of the study and participated in its design. MN participated in the simulation studies. KHJ participated in the design and coordination of the study. All authors read and approved the final manuscript.
